# Relationship between hospital ward design and healthcare associated infection rates: what does the evidence really tell us? Comment on Stiller et al. 2016

**DOI:** 10.1186/s13756-017-0226-8

**Published:** 2017-06-26

**Authors:** Jennie Wilson, Andrew Dunnett, Heather Loveday

**Affiliations:** Richard Wells Research Centre, University of West London, Boston Manor Road, Brentford, Middlesex, TW8 9GB UK

## Abstract

The systematic review published by Stiller et al. in Antimicrobial Resistance & Infection Control in November 2016 concludes that single-patient bedrooms confer a significant benefit for protecting patients from healthcare associated infection and colonization. This conclusion is not substantiated by the evidence included in their review which has been largely drawn from uncontrolled before and after studies in the absence of a transparent assessment of the risk of bias. There are also errors in the analysis of supporting data. Evaluating the specific impact of single rooms on preventing transmission from a sound epidemiological perspective is essential to assure safe and effective care and a clear evidence-base for infection prevention and control advice.

## Letter to Editor, Antimicrobial Resistance & Infection Control

We have read with interest the systematic review by Stiller et al. [1] but are concerned that some of the analyses and subsequent inferences are misleading. We would suggest that their conclusion that single-patient bedrooms confer a significant benefit for protecting patients from healthcare associated infection (HCAI) and colonization is not substantiated by the evidence included in their review.

The methods do not include any detail on the construction of the Forest plots but we would question the value of pooling data from studies that measured different outcomes and, as outlined below, were prone to bias. Of the nine studies included that addressed the question of single rooms, six [2–7] were uncontrolled before and after studies, a design that is recognized to be highly vulnerable to bias [8]. Such bias is a particular problem in the older studies from the 1990s and early 2000s [2–4], which reflect opportunistic evaluations of a move from multi-occupancy to single room accommodation and did not adequately account for differences in case-mix between the two groups. For example, in the study by Ben-Abraham et al. [2] the length of stay in the pediatric intensive care unit (ICU) during the multi-bed period was more than double that in the single room period suggesting a significant difference in case mix that could have affected the key outcomes of ventilator-associated pneumonia (VAP) and urinary tract infection (UTI) that were purportedly associated with room status. Similarly, McManus et al [3] evaluated the impact of moving burn patients from multi-bed to single room accommodation by comparing the rate of bloodstream infections (BSI) post-move (1984 to 1993) with pre-move (1974 and 1983). Endogenously acquired BSI were not distinguished, no adjustment was made for case-mix and there was no consideration of changes in infection control procedures after the move or advances in patient treatment and management over this 20-year period which were highly likely to have influenced the observed reduction in BSI. The more recent study by Ellison et al. [6] attempted to include some element of random assignment to single or multi-bed accommodation and controlled for potential confounding variables. Despite adequate power, this study found no significant difference in acquisition of infection or colonization attributable to room allocation.

It is important to consider aspects of practice that changed with the introduction of single rooms and which may be responsible for reduced transmission rather than the room itself. For example, in the study by Lazar et al. [5] the new single-room unit was less crowded, had more sinks and antiseptic handrub, disposable gloves and other equipment was more accessible, rooms were disinfected after discharge and IV line dressings were changed from gauze to semi-permeable film. All these factors are likely to have had an impact on the occurrence of HCAI regardless of the single room. Improvements in hand hygiene may accompany a move to single rooms, and it is important to acknowledge that this maybe an explanatory factor rather than the physical separation of the patient [6, 9]. In addition, in some studies the change in accommodation was triggered by an outbreak of resistant pathogens and the introduction of other control measures could have explained the results [3, 9].

A further three cohort studies compared acquisition of HCAI in patients admitted to single or multi-bed areas of the same ICU [10, 11] or a ‘control’ ICU [9]. The results of each of these studies has been misrepresented in the Forest plot presented by Stiller et al. Julian et al [10] specifically concluded that there was no significant difference in rate of acquisition of healthcare associated BSI (43% of which were coagulase negative staphylococci and likely to reflect endogenous infection) or MRSA colonization in neonates in single and multi-bed accommodation. The crude data used in their Forest plot is both incorrect and gives the impression of a strongly significant association with single rooms. Other data in the Forest plots includes errors [11] and has been based on crude rates which do not account for the effect of other confounding factors [9, 11].

Finally, several of the included studies have made assumptions that any HCAI is potentially acquired through transmission and therefore single room accommodation could be responsible for their prevention. The epidemiology of these infections is complex and multi-factorial. A BSI can be derived from a variety of primary sources of infection, many endogenous, and as with other outcomes such as catheter-associated urinary tract infections and ventilator-associated pneumonia, they are strongly associated with the duration of device use. It seems unlikely that the use of single room accommodation alone would result in significant improvement in the care of devices.

The use of single room accommodation to manage patients with potentially transmissible infections is a common infection prevention and control strategy but is underpinned by limited evidence of effectiveness [12]. Understanding how single rooms may contribute to interrupting transmission is essential to support their judicious use, as they are a limited resource and their use may also adversely impact the care of some patients [13]. Studies are rarely able to pinpoint the specific effect of the single room accommodation and their use is invariably associated with changes in other critical components of infection control such as hand hygiene and cleaning. Assimilation of evidence from uncontrolled before and after studies in the absence of a transparent assessment of the risk of bias and understanding of the strengths and weakness of the evidence is not helpful in effectively informing guidance or practice [14]. Evaluating the specific impact of single rooms on preventing transmission from a sound epidemiological perspective is essential to assure safe and effective care and a clear evidence-base for infection prevention and control advice.

Yours sincerely,

## Abbreviations

BSI: Blood stream infection
HCAI: Healthcare associated infection
ICU: Intensive care unit
UTI: Urinary tract infection
VAP: Ventilator associated pneumonia (VAP)
